# Does the SARS-CoV-2 Spike Receptor-Binding Domain Hamper the Amyloid Transformation of Alpha-Synuclein after All?

**DOI:** 10.3390/biomedicines11020498

**Published:** 2023-02-09

**Authors:** Yulia Stroylova, Anastasiia Konstantinova, Victor Stroylov, Ivan Katrukha, Fedor Rozov, Vladimir Muronetz

**Affiliations:** 1 Belozersky Institute of Physico-Chemical Biology, Lomonosov Moscow State University, Moscow 119991, Russia; 2 Institute of Molecular Medicine, Sechenov First Moscow State Medical University, Moscow 119991, Russia; 3 Faculty of Biotechnology, Lomonosov Moscow State University, Moscow 119991, Russia; 4 Zelinsky Institute of Organic Chemistry, Russian Academy of Sciences, Moscow 119991, Russia; 5 Chemical Faculty, National Research University Higher School of Economics (HSE), Moscow 101000, Russia; 6 Department of Biochemistry, School of Biology, Lomonosov Moscow State University, Moscow 119991, Russia; 7 HyTest Ltd., 20520 Turku, Finland

**Keywords:** SARS-CoV-2 spike RBD domain, alpha-synuclein, amyloid aggregation, post-COVID-19 neurodegeneration, SARS-CoV-2 vaccine, Parkinson’s disease

## Abstract

Interactions of key amyloidogenic proteins with SARS-CoV-2 proteins may be one of the causes of expanding and delayed post-COVID-19 neurodegenerative processes. Furthermore, such abnormal effects can be caused by proteins and their fragments circulating in the body during vaccination. The aim of our work was to analyze the effect of the receptor-binding domain of the coronavirus S-protein domain (RBD) on alpha-synuclein amyloid aggregation. Molecular modeling showed that the predicted RBD complex with monomeric alpha-synuclein is stable over 100 ns of molecular dynamics. Analysis of the interactions of RBD with the amyloid form of alpha-synuclein showed that during molecular dynamics for 200 ns the number of contacts is markedly higher than that for the monomeric form. The formation of the RBD complex with the alpha-synuclein monomer was confirmed immunochemically by immobilization of RBD on its specific receptor ACE2. Changes in the spectral characteristics of the intrinsic tryptophans of RBD and hydrophobic dye ANS indicate an interaction between the monomeric proteins, but according to the data of circular dichroism spectra, this interaction does not lead to a change in their secondary structure. Data on the kinetics of amyloid fibril formation using several spectral approaches strongly suggest that RBD prevents the amyloid transformation of alpha-synuclein. Moreover, the fibrils obtained in the presence of RBD showed significantly less cytotoxicity on SH-SY5Y neuroblastoma cells.

## 1. Introduction

Numerous data have been accumulated about the effects of coronavirus infection and vaccination by vector and RNA vaccines on the nervous system, from the common complication on the olfactory receptors to a variety of cognitive disorders [1,2,3,4]. However, there is relatively little data about the relationship between coronavirus infection and vaccination and socially significant neurodegenerative diseases, especially Alzheimer’s and Parkinson’s disease, due to the short period since the onset of the coronavirus pandemic [5,6,7,8,9]. The SARS-CoV-2 virus is thought to exacerbate Parkinson’s disease by affecting pathological transformation of alpha-synuclein, stimulation of mitochondrial dysfunction, and depletion of dopamine [9]. Several studies have also emphasized the increased vulnerability of patients with Parkinson’s disease to COVID-19 [10,11].

In order for the coronavirus to invade the cell, proteolysis of the site between the S1 and S2 subunits of the spike protein by cellular proteases, primarily furin, must occur, resulting in the release of the S1 subunit [12]. It has been shown that in mice the S1 protein is able to permeate the blood–brain barrier [13]. At present, the question of the interaction of alpha-synuclein with the full-length S-protein has been analyzed in two papers [14,15], but in the context of the effect on synuclein aggregation, the differences between the full-length S-protein and the RBD-containing S1 protein can be very crucial. There is some evidence from molecular modeling that the RBD fragment released as a part of the S1 subunit of the spike protein during proteolysis can interact with major amyloidogenic proteins alpha-synuclein, the beta-amyloid peptide precursor, or with the peptide itself as well as with the prion protein [16]. During vaccination, both the intact C-protein and its fragments can circulate in the organism, including RBD, formed during proteolysis. Moreover, there are vaccines in which the RBD fragment is the only active component [17,18]. Thus, studying the effect of the RBD fragment on the pathological transformation of alpha-synuclein has implications both for elucidating the mechanisms of synucleinopathies in COVID-19infections and for understanding approaches to prevent undesired effects of vaccination.

Molecular modeling techniques have shown that binding of alpha-synuclein to the RBD domain of the spike protein of coronavirus may occur [15,16]. However, the experimental evidence for this interaction is still not at all clear. An attempt to pull the full-length S-protein expressed in HEK293 cells by immunoprecipitation was unsuccessful, although its co-localization with alpha-synuclein has been shown [15]. In addition, it was shown that the full-length S-protein had no noticeable effect on alpha-synuclein aggregation [14].

It should also be noted that molecular modeling of interactions between the RBD domain spike protein of coronavirus and alpha-synuclein is complicated by the fact that in the monomeric form alpha-synuclein is completely unstructured, while in the oligomeric or fibrillar form it may exist in different conformational states. Currently, there is only one publication whose authors applied protein–protein docking to study the interaction of synuclein with RBD [15]. However, this method does not allow us to consider the conformational dynamics of the proteins that form the complex, and in the case of synuclein this can be very important. To study the structure of potential complexes between synuclein and RBD, it is necessary to use the molecular dynamics approach to investigate the stability of the complexes over time, as well as to clarify the structures of the complexes.

Thus, the aim of this work was to perform both experimental and more advanced molecular modeling of the interaction of the RBD domain of the spike protein of coronavirus with various forms of alpha-synuclein, as well as to test experimentally the possible influence of the individual RBD domain of the spike protein of coronavirus on the amyloid transformation of alpha-synuclein.

## 2. Materials and Methods

### 2.1. Materials

In the current work, we used the following chemicals: Thioflavin T, Congo Red, ANS (1-anilinonaphthalene-8-sulfonic Acid), o-phenylenediamine and MTT (Sigma, Burlington, MA, USA); inorganic salts (Panreac, Barcelona, Spain) and buffers (Amresco, Solon, OH, USA); fetal bovine serum (FBS) (HyClone, Logan, UT, USA); GlutaMAX (Gibco, Waltham, MA, USA); Dulbecco’s Modified Eagle Medium (DMEM), trypsin, and penicillin/streptomycin (Paneco, Moscow, Russia). Human neuroblastoma SH-SY5Y cell culture (ATCC) was kindly gifted by Dr. Irina Naletova (University of Catania, Catania, Italy) [19]. Recombinant human ACE2-Fc (the extracellular domain of human angiotensin-converting enzyme 2 (1-740 a.a. of human angiotensin-converting enzyme 2, UniProtKB Q9BYF1), containing at the C-terminus of Fc fragment of human immunoglobulin G, SARS-CoV-2 Spike RBD (a fragment Arg319-Phe541 of SARS-CoV-2 Spike surface glycoprotein, GenBank: QHD43416.1) and anti-RBD antibodies (5308) were from HyTest LLC (Russia); anti-alpha-synuclein antibodies [LB 509] were from Abcam (Cambridge, UK). HRP-conjugated goat anti-mouse secondary polyclonal antibodies were purchased from Jackson Immunoresearch (West Grove, PA, USA).

### 2.2. Purification of Recombinant Alpha-Synuclein

Full-length wild type alpha-synuclein without additional motifs was expressed in E. coli and purified as previously described [16], with minor modifications. A codon 136 mutation from TAC to TAT (Tyr->Tyr) was introduced into the gene to avoid erroneous inclusion of cysteine in the bacterial expression system [20,21]. In the first step, acid precipitation of unwanted proteins was performed by adding 9% HCl to pH 2.8 in the cell extract and by centrifugation at 15,000× *g* (5 min, 4 °C). The pH of the solution was then quickly corrected to 7.6 using 1 M potassium phosphate solution, pH 11. The pH value of the supernatant was adjusted to 7.6 using 1 M potassium phosphate solution, pH 11. Alpha-synuclein was purified by salting out ammonium sulfate to 40% saturation and stored as a concentrated suspension at 4 °C. The final protein concentration in the suspension was 1.5 mg/mL.

Protein concentration of alpha-synuclein solutions was determined spectrophotometrically at 280 nm, using extinction coefficient for 0.1% solution equal to 0.412.

### 2.3. Preparation of Alpha-Synuclein Fibrils

The ammonium sulfate precipitate of alpha-synuclein was collected by centrifugation and dialyzed against PBS buffer, pH 7.4. The sample was then diluted to a final concentration of 0.4 mg/mL (28 μM) with or without 2.8 or 5.6 μM RBD in the same buffer. Glass tubes (0.3 mL) were used to avoid sorption to plastic, pipetted (0.3 mL) into glass tubes and incubated at 37 °C with constant shaking at 600 rpm for 52 h. During incubation at 37 °C with constant shaking at 600 rpm for 52 h, 10 l aliquots were taken for thioflavin T fluorescence analysis to monitor the formation of amyloid aggregates. Simultaneously, the turbidity of the samples was measured as absorbance at 400 nm to detect the process of protein aggregation.

### 2.4. Thioflavin T Fluorescence Assay

Thioflavin T (ThT) fluorescence assay was performed in 96-well FLUOTRAC 200 black immunology plates (Greiner) using excitation wavelength set to 430 nm and the fluorescence was registered close to its maximum at 485 nm. Aliquots of 10 μL were taken from the tested samples of growing fibrils and added to 100 μL of 35 μM ThT solution. After 10 min of incubation at 20 °C, fluorescence intensity was measured on the plate reader PerkinElmer 2030 Multilabel Reader Victor X5. Each point was obtained by three separate measurements of the same protein sample.

### 2.5. Fluorescence Spectroscopy

Fluorescence spectra were measured using FluoroMax-3 spectrofluorimeter (Horiba Jobin Yvon, Longjumeau, France) in a 100-μL quartz fluorimetric cuvette. The slit widths were both set at 5 nm in emission and in excitation pathways. Tested samples were placed in quartz cuvette with optical pathway of 3 mm. Emission spectra were recorded between 300 nm and 400 nm, every 1 nm, with excitation wavelength set at 295 nm at 20 °C. The fluorescence emission of tryptophanyl residue shows a blue shift when its local environment becomes more hydrophobic.

### 2.6. ANS Fluorescence

For the ANS experiments, 10 μL of tested samples were incubated at 20 °C with a 50-fold molar excess of freshly prepared ANS for 60 min in the dark before the analysis. For the acquisition, the excitation was fixed at 365 nm and the emission was collected between 400 and 600 nm at 20 °C, using a 3 mm path-length cuvette. Spectra of ANS fluorescence were acquired with a FluoroMax-3 spectrofluorimeter (Horiba Jobin Yvon, Longjumeau, France).

### 2.7. Congo Red Binding Assay

Freshly prepared water solution of Congo Red was added to tested samples in PBS buffer, pH 4.0 at a molar ratio of 10:1. Congo Red was incubated for 10 min with protein samples before the measurements. Spectra of Congo Red absorption were acquired using Implen NanoPhotometer^®^ NP80 (Munich, Germany) at 20 °C with a 3 mm path-length cuvette. The buffer spectrum was used as blank and subtracted from all other spectra.

### 2.8. Modified ELISA for Studying Protein–Protein Interactions

Solution of ACE2-Fc (10 µg/mL in 100 µL of PBS) were applied into wells of an ELISA plate. The proteins were adsorbed during 1 h incubation on a shaker at 20 °C, then the plate was washed with PBST (PBS with 0.05 % Tween 20). The next step was the addition of the mixture (10 µg/mL in 100 µL of PBS) of RBD and alpha-synuclein in PBS, previously incubated in 1:1 ratio (1.1 mg/mL of each protein) during 1 h at 20 °C, and incubation for 1 h on a shaker at 20 °C. Then the plate was washed with PBST and incubated with primary monoclonal antibodies against RBD or alpha-synuclein in PBST for 1 h at 20 °C. After washing with PBST, the plate was incubated with peroxidase-conjugated secondary anti-mouse antibodies (1 µg/mL in PBST) for 1 h. After washing, the plate was stained with a solution of o-phenylenediamine with hydrogen peroxide according to the standard procedure. The optical density in the wells was determined at 492 nm using a Stat Fax 2100 plate reader.

### 2.9. Circular Dichroism

Circular dichroism (CD) spectra of 20 μM RBD, 20 μM alpha-synuclein and their equimolar mixture were recorded in the far UV region (190–250 nm) at 20 °C using a 0.1 mm path-length cuvette on Applied Photophysics Chirascan CD spectrometer (Applied Photophysics, Leatherhead, UK). CD spectra were recorded after dialysis against 10 mM potassium phosphate buffer, pH 7.4. Each spectrum was the average of five scans.

### 2.10. Cell Viability of Neuroblastoma SH-SY5Y Cells by MTT-Test

SH-SY5Y cells were grown in DMEM/F12 (PanEco, Moscow, Russia), 10% (*v*/*v*) fetal bovine serum (Hyclone, Logan, UT, USA), 1% (*v*/*v*) L-glutamax (Sigma), and 1% (*v*/*v*) antibiotic solution (penicillin, streptomycin) (PanEco, Moscow, Russia). Cell line was used in experiments at passages 25–28. Cells were maintained at 37 °C in 5% CO_2_, and culture medium was changed every 3–4 days. For cell viability, test cells were seeded at a density of 15,000 cells/well (in 100 μL of medium) onto 96-well cell culture treated plates (Eppendorf, Hamburg, Germany). Experiments were performed 24 h post seeding. Amounts of 0.26 μM RBD, 0.8 μM alpha-synuclein monomers, 0.8 μM alpha-synuclein fibrils or mixture (0.26 μM RBD, 0.8 μM alpha-synuclein) after 24 h of fibrillization under described protocol and further dilution were added. Cells were exposed to proteins for 24 h. Cells exposed to full DMEM/F12 were used as controls. Cell viability was estimated by MTT-reagent, based on the reduction of the tetrazolium dye MTT 3-(4,5-dimethylthiazol-2-yl)-2,5-diphenyltetrazolium bromide to formazan, which can be measured spectrophotometrically [22]. Cell medium was changed for DMEM/F12 with tetrazolium dye (final concentration 0.375 mg/mL) and after 4 h of incubation cells were dissolved in 100 mL DMSO. After 10 min at 37 °C absorbance at 570 nm was registered (VersaMax microplate reader, Molecular Devices, San Jose, CA, USA). The reference wavelength at 630 nm was used.

### 2.11. Docking and Molecular Dynamics Simulations

Structures of alpha-synuclein bound to sodium dodecyl sulfate micelles (PDB ID: 1XQ8) and alpha-synuclein fibril obtained by solid state NMR (PDB ID:2N0A) were used for modelling. Protein–protein docking of SARS-CoV-2 S-RBD (PDB ID: 6M0J) with alpha-synuclein was performed on the HDOCK server (http://hdock.phys.hust.edu.cn/, accessed on 11 April 2022), which is based on a hybrid algorithm of template-based modeling and ab initio free docking [23]. The binding modes of macromolecules were evaluated visually using VMD and by the value of binding energy. Then, the interactions within protein complexes were analyzed through the PDBSUM server (http://www.ebi.ac.uk/pdbsum, accessed on 19 April 2022), which is a web server that provides structural information including protein secondary structure, protein–ligand, and protein–protein interactions [24]. Dissociation constant (Kd) of a putative aSyn-RBD complex was estimated using the PPA-Pred server [25].

Molecular dynamics simulations were carried out using the GROMACS 2018 simulation package [26]. Intramolecular interactions are described for the fibril by the CHARMM 36m all-atom force field [27] and the TIP3P water model [28]. Prior to the production MD, each model was energy minimized using the steepest descent algorithm, and equilibrated for 200 ps in an NVT ensemble of 310 K, and then for 200 ps in an NPT ensemble at 310 K and 1 atm, with the heavy atoms restrained with a force constant of 1000 kJ mol^−1^ nm^−2^.

Production MD simulations were commenced from the final frame of equilibration trajectory and run at 310 K and 1 atm. Thermal stabilization was achieved by a v-rescale thermostat with a coupling constant of 0.1 ps, and the pressure was kept constant using the Parrinello–Rahman barostat with a relaxation time of 2 ps. Rigidity of water molecules was kept using the SETTLE algorithm 28, and other covalent bonds involving hydrogen atoms were restrained at their equilibrium lengths using LINCS29 algorithm, with a time step of 2.

Long-range electrostatic interactions were computed with the Particle-mesh Ewald approach, using cutoff of 12 Å and a Fourier grid spacing of 1.6 Å. Short-range van der Waal interactions were truncated at 12 Å, with smoothing starting at 10.5 Å.

## 3. Results

### 3.1. Docking and Molecular Dynamics of Protein–Protein Interaction between SARS-CoV-2 Spike Receptor-Binding Domain (SARS-CoV-2 RBD) and Alpha-Synuclein (SYN)

**Figure 1:**
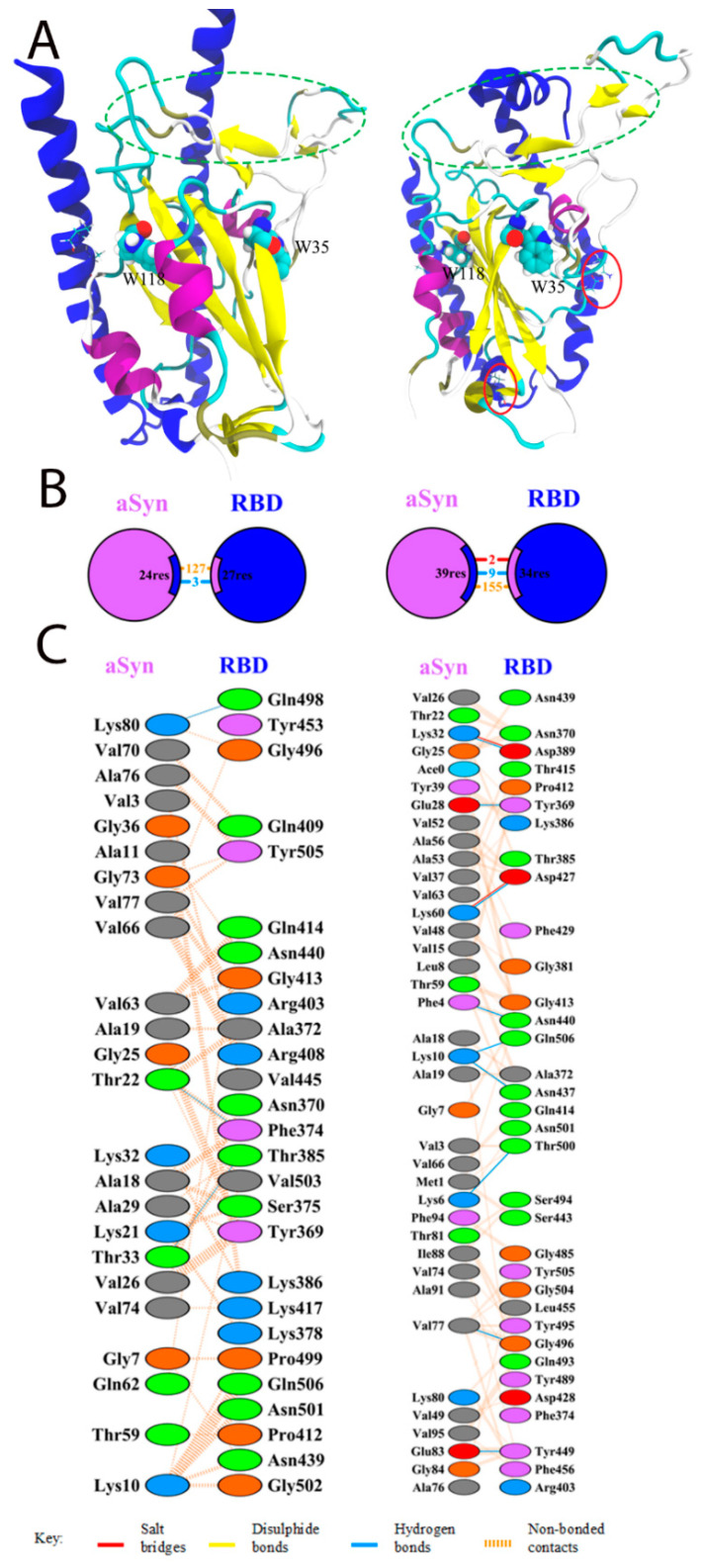
Interaction of SARS-CoV-2 RBD with alpha-synuclein by docking (**left**) or after 100 ns of molecular dynamics (**right**). (**A**) General view of binding. Alpha-synuclein is stained blue. RBD: α-helices are stained violet; β-sheets are yellow; the unstructured part is cyan. Tryptophan residues are highlighted on the RBD; the interaction site with ACE2 is highlighted with green ovals. On the alpha-synuclein as Licorice, residues within 5 Å of tryptophan residues are marked. Red ovals indicate the salt bridges formed during the dynamic simulation. (**B**) Schematic representation of the interaction of RBD with alpha-synuclein. Numbers indicate the amount of salt bridges (red), non-bonded contacts (orange), and hydrogen bonds (blue) that were discovered by docking (**left**) and molecular dynamics (**right**). (**C**) Residues involved in the interaction. (**left**): docking; (**right**): molecular dynamics.

The appearance of protein–protein interactions is the first and key step for many biological interactions involved in cell signaling, immunity, and cellular transport [29]. Molecular modeling techniques are well established for studying protein structure and ligand activity [30,31]. Potential interactions between the RBD domain of SARS-CoV-2 spike protein and alpha-synuclein were studied using the HDOCK server. Model 1 with the highest docking energy and the lowest RMSD of the ligand was chosen. For this model, a molecular dynamic trajectory of 100 ns was performed to clarify the structure of the complex. The results of the docking and molecular dynamics are shown in Figure 1. The PDBSum server was used to determine the interacting residues of protein complexes; such surfaces and residues are shown in Figure 1.

Docking results showed that the interaction between alpha-synuclein and RBD was mediated by three hydrogen bonds via Lys21, Lys80, and Thr22 residues to Val503, Gln498, and Phe 374 of the RBD (Figure 1B,C) and 127 unlinked contacts, which corresponds essentially to the result described by Wu et al. [15]. Interestingly, after molecular dynamics, salt bridges between Lys32 and Lys60 with Asp389 and Asp427 RBD residues, which were absent before, appeared in the structure of the complex. The number of hydrogen bonds and nonpolar contacts increased to 9 and 155, respectively.

The binding affinity of the docking structures, represented by the dissociation constant (Kd), was obtained using PPA-Pred [25]. The predicted value of the dissociation constant was 7.26 × 10^−10^ M, which is about two orders of magnitude lower than previously reported (1.46 × 10^−8^ M) [25].

Thus, we can say with reasonable certainty that an interaction between RBD and the monomeric form of alpha-synuclein is possible.

**Figure 2:**
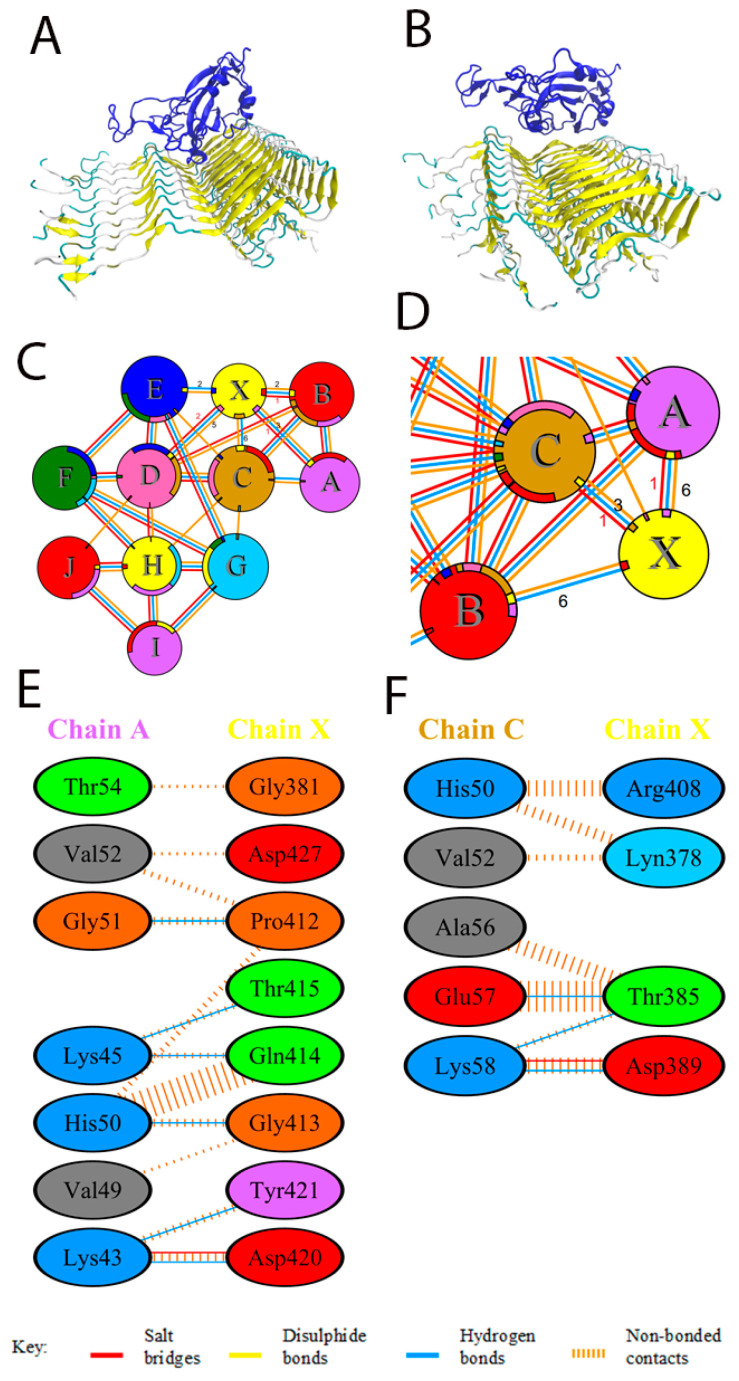
Interaction of SARS-CoV-2 RBD with the oligomer of alpha-synuclein by docking (**left**) or after 200 ns of molecular dynamics (**right**). Chains from A to I correspond to alpha-synuclein; chain X corresponds to RBD. (**A**,**C**) General view of binding and scheme of interactions according to docking results. Alpha-synuclein is colored yellow; RBD is colored blue. (**A**,**B**) General view. (**C**,**D**) Schematic representation of the interaction of RBD with alpha-synuclein after molecular dynamics. (**E**,**F**) Residues with the most significant contribution to the binding according to calculations. (**left**) docking; (**right**) molecular dynamics.

Potential interactions between the RBD domain of the SARS-CoV-2 S-protein and the alpha-synuclein oligomer/fibril (PDB ID 2n0a) were studied using the HDOCK server. Model 2 was chosen, featuring both high interaction energy and the involvement of the RBD site, which is characterized by interaction with ACE2, in the protein–protein contact. For this model a molecular dynamic trajectory of 200 ns was calculated to clarify the structure of the complex. The results of docking and molecular dynamics are shown in Figure 2. The PDBSum server was used to determine the interacting residues of the protein complexes. The interacting surfaces and binding residues are shown in Figure 2.

The docking results showed that RBD is able to form a fairly tight contact with the oligomer of alpha-synuclein, which involves the site responsible for the interaction with ACE2. However, as a result of molecular dynamics, a change in the orientation of RBD relative to the micelle is observed, which leads to a shift of the contact region to the lateral surface of the receptor fragment. This shift, however, does not hamper the binding to the full-length spike protein. Two salt bridges and 15 hydrogen bonds are observed in the resulting structure, which exceeds the parameters obtained for the alpha-synuclein monomer. Lys43 and Lys58 residues are involved in the formation of salt bridges on the alpha-synuclein side, and Asp389 and Asp420 residues on the RBD side (numbering shifted).

It is particularly noteworthy that the oligomer/fibril structure of alpha-synuclein is considerably compacted with an increase in the number of interchain interactions during the process of molecular dynamics (Figure 2E,F).

### 3.2. Interaction of the Monomeric Form of Alpha-Synuclein with the SARS-CoV-2 RBD

**Figure 3:**
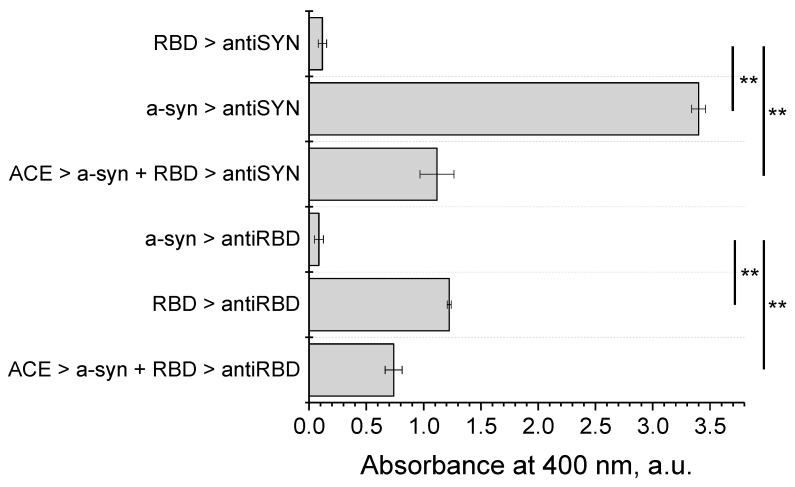
ACE2 interacts with pre-incubated SARS-CoV-2 RBD and alpha-synuclein by modified ELISA. Pre-incubated alpha-synuclein and SARS-CoV-2 S1 RBD during 1 h at 20 °C was applied to ACE2-Fc and detected by anti-RBD (5308) and anti-alpha-synuclein (LB509) antibodies. The poles represent the mean value of three independent measurements and error bars show the standard deviation. P-value represents results of one-way ANOVA; ** *p* < 0.01.

First, the possibility of binding of the monomeric form of alpha-synuclein to RBD was checked. For this purpose, an immunochemical method was used to identify the complex of the two proteins. To obtain the complex, the monomeric form of alpha-synuclein was incubated for one hour with RBD and then injected into wells of a plate containing pre-sorbed ACE2-Fc, which binds to the RBD of spike protein upon virus entry into the cell. This approach mimics immunoprecipitation, in which ACE2-Fc acts as an antibody by interacting with RBD. As follows from the data in Figure 3, using specific monoclonal antibodies to these two proteins, we showed that not only RBD but also the associated alpha-synuclein is sorbed onto ACE2-Fc, indicating formation of a complex between the two proteins.

**Figure 4:**
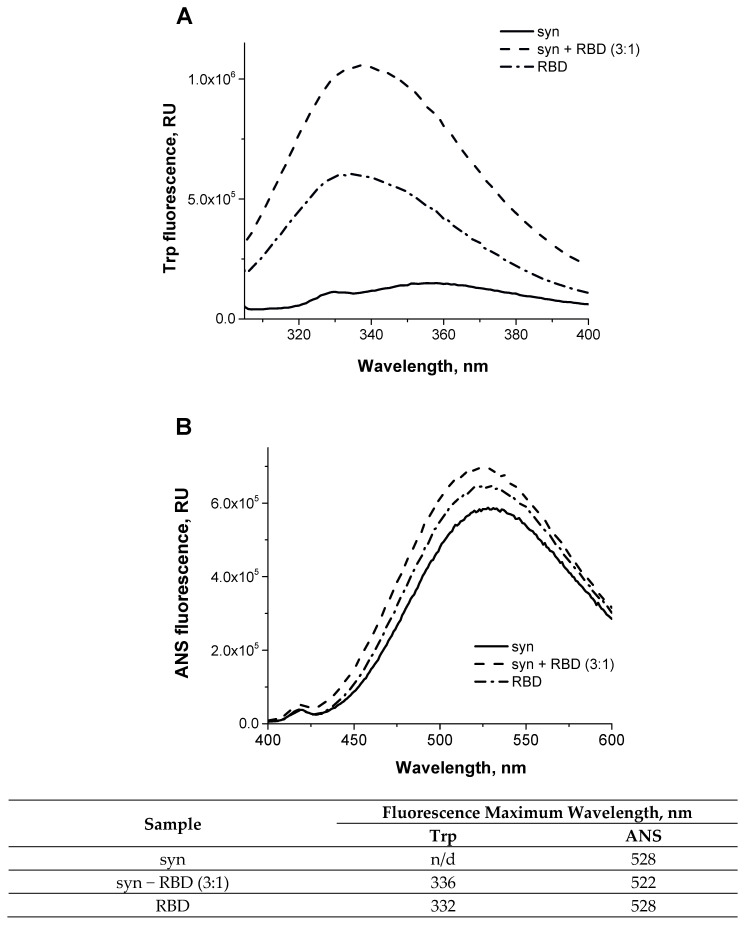
The structural change of the SARS-CoV-2 RBD observed in the presence of alpha-synuclein by Trp (**A**) and ANS (**B**) fluorescence. Trp (**A**) and ANS (**B**) fluorescence emission spectra of 5.6 µM SARS-CoV-2 RBD (dot-dashed line), 28 µM alpha-synuclein (solid line), 28 µM alpha-synuclein mixed with 9.3 µM SARS-CoV-2 RBD (molar ratio 3:1, dashed line). Please note that alpha-synuclein does not contain tryptophans (solid line).

**Figure 5:**
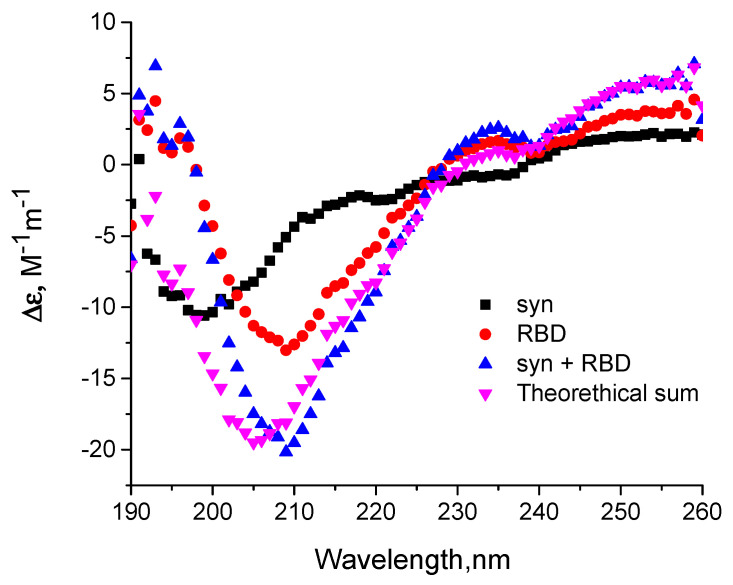
Influence of alpha-synuclein on circular dichroism spectra of SARS-CoV-2 RBD. CD spectra of 20 µM alpha-synuclein (squares) and 20 µM SARS-CoV-2 RBD (circles), equimolar mixture of Syn and RBD (blue triangles pointed up), and theoretical sum (violet triangles pointed down) are shown. CD spectra of the investigated samples were recorded after buffer exchange to 10 mM potassium phosphate buffer, pH 7.4. Each spectrum is an average of five records.

To confirm the formation of a complex between the monomeric form of alpha-synuclein and RBD, we also analyzed the change in the spectral characteristics of the proteins after their mixing. Figure 4A show the fluorescence spectra of RBD’s own tryptophans before and after its binding to alpha-synuclein, which has no tryptophans. The shift in the fluorescence spectrum of RBD tryptophans may indicate their partial transition from the hydrophobic to the aqueous environment. The increased intensity and shift of the fluorescence maximum of 1,8-ANS interacting with hydrophobic sites when it binds to the complex of two proteins (Figure 4B) indicates a change in the conformation of alpha-synuclein and/or RBD during their interaction. However, the secondary structure of the proteins does not change during complex formation. This is evidenced by the CD spectra shown in Figure 5. As follows from these data, summing the spectra of the original proteins gives a spectrum characteristic of their complex.

Thus, it was shown that monomeric alpha-synuclein is bound by RBD, causing changes in some spectral properties of the proteins, indicating a change in their conformation while keeping the secondary structure intact.

### 3.3. Effect of SARS-CoV-2 RBD on the Amyloid Transformation of Alpha-Synuclein

**Figure 6:**
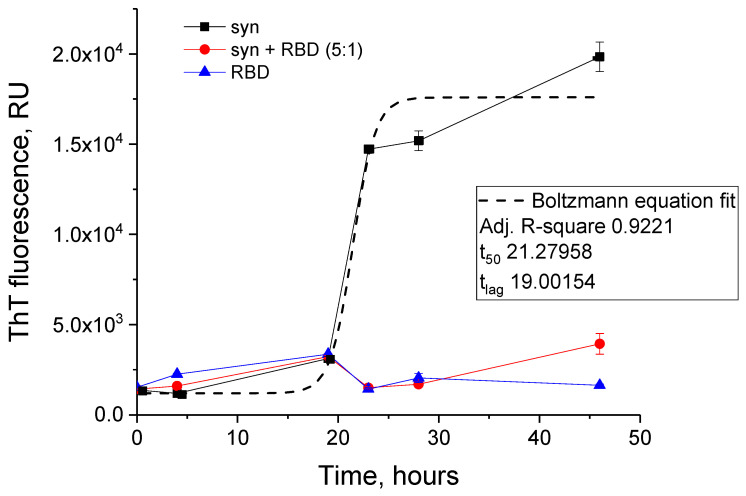
ThT fluorescence fibril formation kinetics of alpha-synuclein in the absence and presence of SARS-CoV-2 RBD. ThT fluorescence of 5.6 µM SARS-CoV-2 RBD (triangles), 28 µM alpha-synuclein (squares), 28 µM alpha-synuclein and 5.6 µM SARS-CoV-2 RBD (molar ratio 5:1, circles).

**Figure 7:**
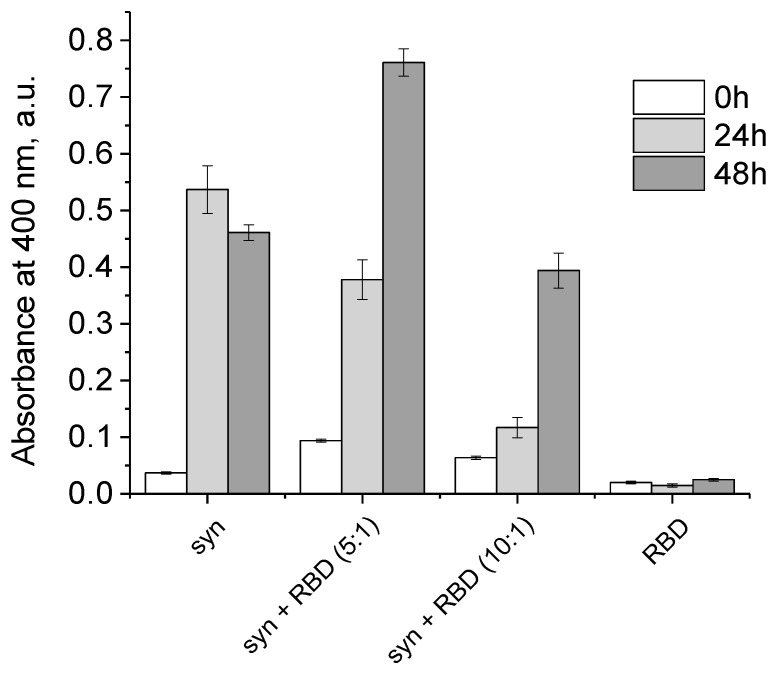
Aggregation of the alpha-synuclein, fibrillized in the presence of SARS-CoV-2 RBD. Absorbance at 400 nm of 5.6 µM SARS-CoV-2 RBD, 28 µM alpha-synuclein, 28 µM alpha-synuclein and 5.6 µM SARS-CoV-2 RBD (molar ratio 5:1), and 28 µM alpha-synuclein and 2.8 µM SARS-CoV-2 RBD (molar ratio 10:1) for 0 h (white), 24 h (light gray), and 48 h (dark gray).

As results of thioflavin T fluorescence on Figure 6 clearly demonstrate, RBD of the spike protein completely blocks the amyloid transformation of alpha-synuclein. However, the RBD does not prevent disordered aggregation of alpha-synuclein, based on changes in the turbidity of the solution of the mixture of the two proteins (Figure 7). Some differences in the turbidity of the mixture of the two proteins probably depend both on their total concentration and on the characteristics of aggregation at different ratios of alpha-synuclein to RBD.

**Figure 8:**
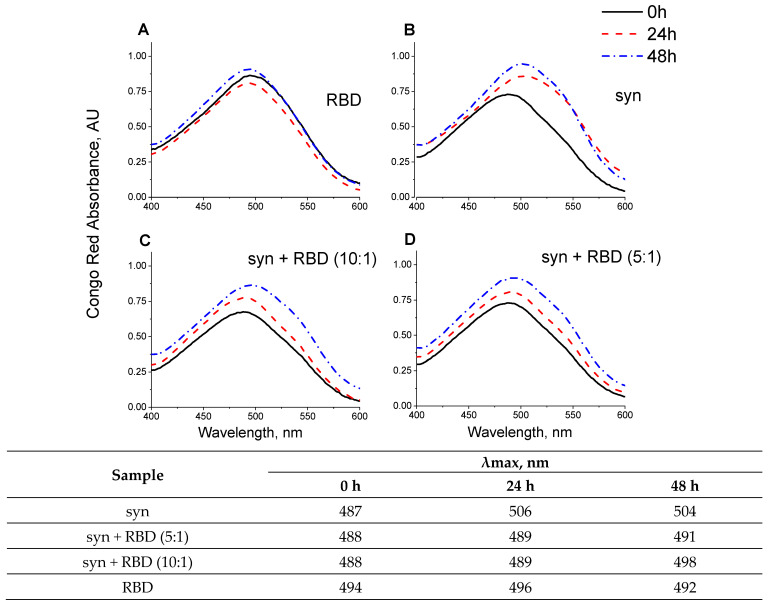
Congo Red absorbance changes of the alpha-synuclein, fibrillized in the presence of SARS-CoV-2 RBD. Congo Red absorbance spectra of SARS-CoV-2 RBD (**A**), 28 µM alpha-synuclein (**B**), 28 µM alpha-synuclein and 5.6 µM SARS-CoV-2 RBD (molar ratio 5:1, (**C**)), 28 µM alpha-synuclein and 2.8 µM SARS-CoV-2 RBD (molar ratio 10:1, (**D**)) for 0 h (solid line), 24 h (dashed line), and 48 h (dash-dotted line).

**Figure 9:**
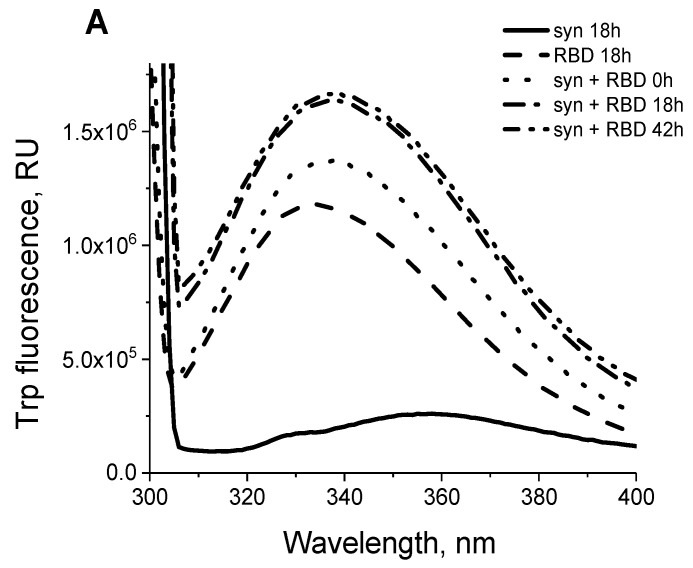

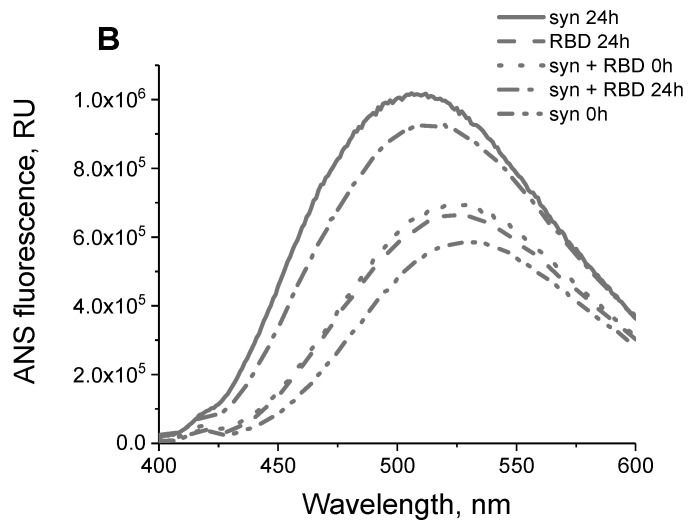
Structural changes of the SARS-CoV-2 RBD observed under fibrillization of alpha-synuclein by Trp and ANS fluorescence. Trp (**A**) and ANS (**B**) fluorescence emission spectra SARS-CoV-2 RBD (dashed line), 28 µM alpha-synuclein and 5.6 µM SARS-CoV-2 RBD (molar ratio 5:1) for 0 h (dash-dotted line) and 18–24 h (dotted line). Alpha-synuclein does not contain tryptophans (solid line for 18-24 h and dash-double-dot line for 0 h).

The prevention of amyloid transformation of alpha-synuclein in the presence of the RBD is also evidenced by the spectra of Congo Red shown in Figure 8. During the interaction of Congo Red with alpha-synuclein fibrils, a shift of the dye absorption spectrum by almost 20 nm is observed. The spectra of the dye upon addition of the aggregates obtained in the presence of the RBD practically do not change. RBD also prevents changes in the ANS fluorescence spectra characteristic of dye interactions with amyloid forms of alpha-synuclein, primarily the amplitude (Figure 9). The spectra of tryptophan residues of the RBD fragment in interaction with monomeric forms and in aggregates are almost identical (Figure 4 and Figure 9).

### 3.4. Changes in Cytotoxicity of Synuclein Fibrils during Their Formation in the Presence of RBD

**Figure 10:**
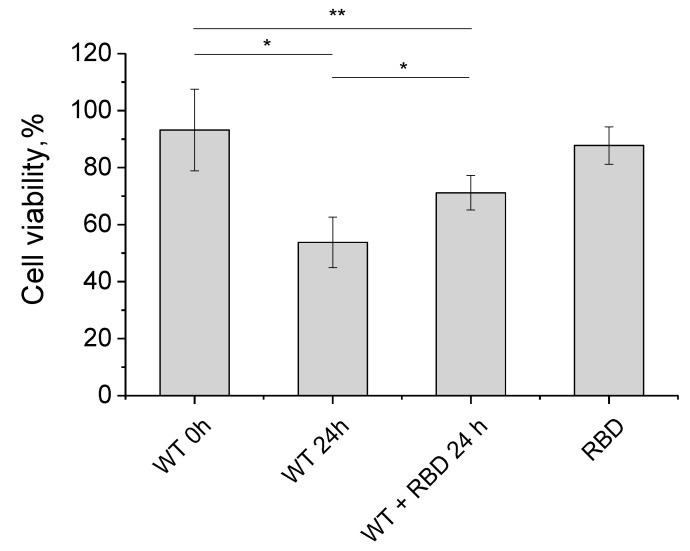
The effect of the alpha-synuclein fibrils, obtained in the presence of SARS-CoV-2 RBD, on neuroblastoma SH-SY5Y viability by MTT-test. The 28 µM alpha-synuclein fibrils (with or without 5.6 μM RBD) were produced for 24 h (as described in the Materials and Methods section). After dilution, 0.8 μM synuclein, 0.26 μM RBD, and 0.8 μM synuclein with 0.26 μM RBD (molar ratio 3:1) were added to SH-SY5Y neuroblastoma cells, and cell viability was determined after 24 h using the MTT test. *p*-value represents results of one-way ANOVA; * *p* < 0.05; ** *p* < 0.01.

The influence of RBD on the formation of alpha-synuclein amyloid fibrils was also tested in cytotoxicity experiments. Alpha-synuclein fibrils were produced by incubating 28 μM monomeric alpha-synuclein for 24 h at pH 4.0 as well as by incubating a mixture of alpha-synuclein monomers in the presence of 5.6 μM RBD. The resulting fibril preparations were diluted in DMEM/F12 to final concentrations of 0.8 μM for alpha-synuclein and 0.26 μM for RBD. The fibril solution was added to the neuroblastoma cells and cell viability was assessed after 24 h of incubation. As follows from the Figure 10, amyloid fibrils reduce cell viability by 50 percent, while RBD itself has no cytotoxic effect. The cytotoxic effect of alpha-synuclein fibrils formed in the presence of RBD is significantly lower than that of conventional alpha-synuclein fibrils, indicating their diminished amyloidogenic properties.

## 4. Discussion

The data of our molecular simulations are in good agreement with the experiments performed. According to our molecular simulation, the binding site of the RBD fragment to alpha-synuclein is nearly almost unaffected by the regions involved in the interaction with the cellular ACE2 receptor (labeled with ovals in Figure 1A). We tested this interaction in an experiment by binding a mixture of RBD fragment and alpha-synuclein to immobilized ACE2, followed by the detection of the developed complex with anti-RBD and anti-Syn antibodies. Indeed, judging from the results of the experiment, the binding of alpha-synuclein to RBD does not interfere with the interaction of RBD with ACE. Moreover, according to docking, two tryptophan residues in the RBD molecule are located quite close to the alpha-helices of alpha-synuclein (marked in Figure 1A); therefore, these residues can serve as sensitive indicators of interaction, as we see in the observed change in fluorescence in the experiments.

In contrast to amorphous aggregation, the formation of amyloid is a complex organized process with a number of specific features, such as changes in the initial conformation of the protein, ability of “seeding”, formation of intermediate oligomers, and growth of an unbranched fibril in a strictly determined mode. Therefore, among the possible explanations for how amyloid aggregation is inhibited when interacting with RBD, we can assume the difficulty of changing conformation during binding, inhibition of the formation of intermediate oligomers, and “capping” of the fibril ends to prevent further growth. Indeed, interaction with other molecules may both prevent [32,33] and stimulate amyloid transformation of alpha-synuclein [34,35].

The S-protein was chosen for immunization because it is a surface penetration protein abundantly present in the viral particle, and a strategy aimed at producing neutralizing antibodies that prevent the virus from penetrating cells is most effective in terms of preventing/suppressing disease. The question of whether circulating S-protein and its fragments will influence the pathological transformation of amyloid proteins seems important, since this interaction might lead to side effects of vaccination. Evidence of worsening the course of infection in Parkinson’s disease patients [10] indicated the possibility of stimulation of amyloid transformation of alpha-synuclein by coronavirus proteins. However, in vitro it has been shown that full-length coronavirus S-protein did not bind alpha-synuclein during immunoprecipitation and had no effect on its aggregation [15]. Yet, given the release of the S1 subunit during proteolysis of the protein in the cell and the use of truncated proteins with the RBD fragment for coronavirus vaccine development, we performed a study of the RBD fragment. It was shown that the RBD fragment not only does not stimulate amyloid transformation of alpha-synuclein, but instead prevents it by stimulating amorphous aggregation. The combination of these data shows that the use of vaccines causing RBD of S-protein to circulate in the blood should not stimulate the formation of amyloid aggregates, at least in synucleinopathies. However, such stimulation can occur with the COVID-19 disease itself or with the use of whole virus particles containing both S- and N-proteins. At the same time, work has appeared showing that N-protein causes amyloid transformation of alpha-synuclein [14]. So we can presume that the pro-Parkinson manifestations of SARS-CoV-2 could be attributed to the action of N- but not S-protein.

Given that Parkinson’s disease and other amyloid diseases usually have a very prolonged (years!) preclinical stage, when vaccinating with an agent capable of contacting amyloidogenic proteins, the effect of vaccines on the pathological transformation of proteins must be taken into account. At the same time, different types of vaccines can have different effects on the development of amyloidosis. For example, in the case of Parkinson’s disease, the RBD fragment of the S-protein may even act as an anti-amyloid agent, whereas N-protein-containing vaccines, on the contrary, may provoke the disease. It is likely that clinical trials of new vaccines should include similar studies with major amyloidogenic in vitro proteins, since traditional animal and human safety trials are not conducted for a sufficient amount of time, and the consequences can be more than serious. In this case, the in vitro approach allows us to simulate the accelerated interaction of proteins and the possibility of amyloid transformation. Of course, based on the data obtained on the effect of only one RBD fragment on the pathological transformation of alpha-synuclein, it is difficult to talk about the possible mechanisms of the development of postvaccine complications. However, these data may already be useful for the development of vaccines against this infection. At the current stage of development of this field of science, in our opinion, it is preferable to use strictly defined proteins or their fragments produced by both RNA and vector vaccines for vaccination in order to reduce the possibility of unpredictable consequences due to the circulation of a whole set of proteins in the body. Moreover, the use of vaccines with only the RBD fragment, which does not stimulate the amyloid transformation of alpha-synuclein, as opposed to the full-length S-protein capable of forming amyloidogenic proteolytic fragments, may be sufficient.

Nystrom et al. recently showed that the S-protein contains several peptides capable of forming amyloid fibrils, and its coincubation with protease neutrophil elastase can result in the accumulation of one of the amyloidogenic peptides 194–203 [36]. As the amino acid sequence of RBD (319–541) does not include those S-protein peptides (192–211, 601–620, and 1166–1185) for which maximum amyloid criteria were shown, as well as amyloidogenic peptide 194–203 derived from cleavage of full-length S-protein by protease neutrophil elastase. This, in turn, indicates the likely benefits and safety of using RBD-based vaccines.

## 5. Conclusions

Using molecular modeling methods and biochemical approaches, we showed that the RBD of the coronavirus S-protein is able to interact with both monomeric and amyloid forms of alpha-synuclein. According to the molecular modeling data, the binding to the amyloid of alpha-synuclein is stronger than to the original monomer of the protein. It is also likely that this interaction makes further formation of amyloid fibrils more difficult. Although aggregates do form in the presence of RBD, they not only have less pronounced amyloid properties but also possess less cytotoxicity. In regards of post-COVID-19 neurodegeneration and provocation of Parkinson’s disease manifestation, our results refute the initial assumption that the RBD of S-protein provokes the amyloid transformation of alpha-synuclein.

Thus, our results support the possibility of using the RBD of S-protein for vaccination and immune response production as sufficiently safe in the context of the development of neurodegenerative complications. At the very least, the presence of RBD in the body should not provoke the development of synucleinopathies.

## Data Availability

Not applicable.
